# Differential effects of 3-nitrooxypropanol supplementation on milk fatty acid profiles in 3 different dairy breeds

**DOI:** 10.3168/jdsc.2024-0656

**Published:** 2024-12-16

**Authors:** Gayani M.S. Lokuge, Nicolaj I. Nielsen, Morten Maigaard, Peter Lund, Lotte Bach Larsen, Lars Wiking, Nina Aagaard Poulsen

**Affiliations:** 1 Department of Food Science, Aarhus University, Agro Food Park 48, DK-8200 Aarhus N, Denmark; 2 SEGES Innovation P/S, Agro Food Park 15, DK-8200 Aarhus N, Denmark; 3 Department of Animal and Veterinary Sciences, Aarhus University, AU Viborg–Research Centre Foulum, Blichers Allé 20, DK-8830 Tjele, Denmark

## Abstract

3-Nitrooxypropanol (3-NOP) is a promising and approved antimethanogenic feed additive in dairy farming. The objective of this study was to evaluate breed-specific variation in milk fatty acids (FA) following 3-NOP supplementation. Milk FA composition was analyzed from 3 studies using Danish Holstein (DH), Danish Jersey (DJ), and Danish Red (DR) dairy cows respectively, fed control or 3-NOP supplemented diets. The 3-NOP was supplemented at a dose of 60 mg/kg feed DM. Bulk milk samples were analyzed for milk FA by GC flame ionization detection. The 3-NOP supplemented diets increased the proportion of short-chain fatty acids (SCFA) and decreased the proportion of C16:0 in the milk of DH cows. A similar tendency in milk FA composition induced by 3-NOP was observed in DJ cows; however, the extent of these changes was less pronounced, and no changes were observed in milk FA from DR cows. Indirect comparison among the breeds showed a higher proportion of SCFA in milk from DJ cows, a higher proportion of medium-chain FA in DR cows and a higher proportion of long-chain FA in DH cows. The effect of 3-NOP on milk FA composition appears to vary between breeds; however, this variation may also be influenced by differences in feeding regimens between breeds, making further studies worthwhile to better understand these interactions.

## Highlights

• 3-NOP increases short-chain fatty acids in milk from DH cows by 9.18%.
• 3-NOP increases short-chain fatty acids in milk from DJ cows by 4.13%.
• 3-NOP increases short-chain fatty acids in milk from DR cows by 2.66%.
• The effect of supplementing 3-NOP on milk FA composition is breed specific.

## Graphical Abstract

**Figure:**
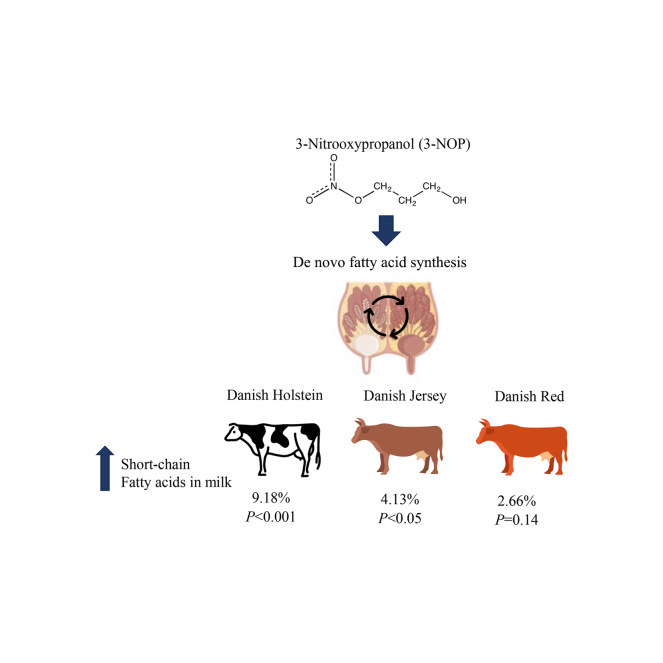
**Summary:** 3-Nitrooxypropanol (3-NOP) is a synthetic compound used to reduce methane emissions from dairy cows. Its effect on cow performance and milk composition has gained increasing interest in recent research studies. In the present study, the effect of 3-NOP on milk fatty acids (FA) was investigated in 3 different dairy breeds, namely Danish Holstein (DH), Danish Jersey (DJ), and Danish Red (DR). The results showed that feeding 3-NOP significantly increased the concentration of short-chain FA in the milk of DH cows to a greater extent than in DJ cows, whereas no significant changes were observed in DR cows. In conclusion, the results indicated that the effect of 3-NOP on milk FA composition is breed specific, likely due to inherent differences in de novo FA synthesis among breeds.

3-Nitrooxypropanol (**3-NOP**) has recently attracted great research interest in the dairy sector due to its ability to reduce methane (CH_4_) emissions from ruminants. The mode of action of 3-NOP involves targeting and inhibiting methyl-coenzyme M-reductase, which is responsible for the final step in the CH_4_ synthesis pathway (Duin et al., 2016). Recent studies have reported an average of around 30% reduction in CH_4_ yield (g CH_4_/kg of DMI) from dairy cows upon feeding 3-NOP-supplemented diets (Melgar et al., 2021; Kebreab et al., 2023; Maigaard et al., 2024). However, the efficacy of 3-NOP depends on the dose and duration of 3-NOP supplementation, the composition of the basal diet, and the presence of other CH_4_-inhibiting compounds in the diet (Dijkstra et al., 2018; Kebreab et al., 2023; Maigaard et al., 2024; van Gastelen et al., 2024). Even though 3-NOP does not add any nutritional value to the cows' diet, this compound could potentially affect DMI, nutrient digestibility, ruminal fermentation, pH, and microbiota of the cow (Veneman et al., 2015; Lopes et al., 2016; Melgar et al., 2020a). These effects could subsequently affect the nutrient availability for milk synthesis or feed-derived nutrients drawn into milk, ultimately affecting milk composition. In previous studies, 3-NOP has been shown to increase milk fat content (Lopes et al., 2016; Melgar et al., 2020b, 2021) and modify milk fatty acid (**FA**) composition (Melgar et al., 2021; van Gastelen et al., 2022; Lokuge et al., 2024b).

Milk FA composition is very complex containing ∼400 individual FA. However, only around 15 of these are found in considerable amounts in bovine milk (McSweeney et al., 2020). Milk FA with even chain length below C16:0 and approximately half of C16:0 are synthesized de novo within the mammary gland using acetate and BHB originating from ruminal fermentation as substrates. Long-chain FA (>C16; **LCFA**) and the rest of C16:0 are either absorbed directly from the feed, originate from microbial de novo synthesis, or mobilized body fat (Grummer, 1991). Genetic variation in FA can be exhibited among breeds. Milk fat from Jersey cows has higher proportions of short- and medium-chain FA (**SCFA** and **MCFA**, respectively) and lower proportions of C18:1 FA than Holstein cows (Beaulieu and Palmquist, 1995; White et al., 2001). Studies on Danish dairy herds have reported similar variations in milk FA composition between Danish Holstein (**DH**) and Danish Jersey (**DJ**) cows (Poulsen et al., 2012). The cows' capacity for de novo FA synthesis in the mammary gland, rumen biohydrogenation (**BH**) and desaturase activity can partly explain some of these differences (Larsen et al., 2012; Weisbjerg et al., 2013). Given that genetic and environmental factors influence milk FA composition, we hypothesized that DH, DJ, and Danish Red (**DR**) cows respond differently in terms of the changes in milk FA composition when including 3-NOP in the diet.

The data included in this article were from 3 different studies. The experimental design of the first study was explained in detail by Lokuge et al. (2024a). In brief, 48 DH cows were blocked according to days in milk and parity in a randomized block design. The cows in the control group were fed basal diets without 3-NOP and cows in the 3-NOP group were fed basal diets supplemented with 3-NOP at the dose of 60 mg/kg feed DM. Both groups received experimental diets for 12 consecutive weeks before milk sampling. The basal diet consisted of grass-clover silage, corn silage, spring barley, rapeseed cake, rapeseed meal, sugar beet pulp, and mineral supplements. This experiment was conducted at the Department of Animal and Veterinary Sciences at Aarhus University (Tjele, Denmark) from April to June 2023. For the 2 other breeds (DJ and DR), all the cows received basal diets supplemented with 3-NOP (60 mg/kg feed DM) during period I, and then they received the basal diets without 3-NOP (control) in period II. In the study with DJ cows, milk was collected from 2 commercial farms (farm A and B) in Denmark. There were 180 lactating cows on farm A and the basal diet was composed of corn silage, grass silage, rapeseed meal, rapeseed cake, palm fat (pure C16:0), and minerals. The period I of this study was conducted from November 2022 until March 2023 and period II was in April 2023. Farm B had 650 lactating cows, and their basal diet consisted of corn silage, grass silage, rapeseed meal, rapeseed cake, rye, palm fatty acids distillate (C16:0 and C18:0), and minerals. Period I was from December 2022 to March 2023 and period II was in April 2023. The study with DR cows was also performed at a Danish experimental farm (Assendrup Hovedgaard, Haslev, Denmark) with 280 lactating cows. The basal diet included maize silage, grass silage, wheat straw, NaOH-treated wheat, rapeseed meal, rapeseed cake, and minerals. Each experimental period lasted 30 d, and the experiment was conducted from January to March 2022. The crude fat content of the basal diets in studies was 41, 56, 58, and 30 g/kg DM, respectively. All the studies were conducted in indoor housing system. Cows were milked twice daily and bulk milk samples from each treatment group were collected. The FA analysis in milk fat was done by GC as described by Lokuge et al. (2024b).

Milk FA data from DH and DR cows were analyzed using a general linear model (**GLM**) with the fixed effect of treatment (control and 3-NOP), using R software (R 4.0.5; https://www.r-project.org). For DJ cows, the fixed effect of treatment, the fixed effect of the farm, and the interaction effect between the farm and treatment were included in GLM. Least squares means were calculated, and multiple comparisons were conducted using the Tukey post hoc test. Differences were considered significant if *P* < 0.05. Multivariate data analysis was done using SIMCA17 (Sartorius Stedim Data Analytics AB). The results were interpreted carefully due to the presence of confounding effects between breed and herd.

The addition of 3-NOP markedly affected the milk FA composition of DH cows (Table 1). In DH cows, concentrations of C4:0, C6:0, C8:0, C10:0, and C11:0 were greater in cows fed 3-NOP diets compared with those fed control diets, resulting in a greater total SCFA content in milk of cows receiving the 3-NOP diets. Furthermore, the concentrations of some MCFA (C12:0, C13:0, C14:0, C15:0, and C17:0), except C16:0 were greater in DH cows fed 3-NOP diets than control diets. Regardless of these increments, the total MCFA tended to be lower in milk from 3-NOP fed cows as C16:0 concentration was lower in milk from those cows. In addition, the concentrations of C18:1 *cis*-9, CLA *cis*-9, and *trans*-11 were decreased, and concentrations of C18:0, C18:2 n-6 *cis*, and C18:3 n-3 were increased by 3-NOP supplementation. However, the total concentration of LCFA was not altered by 3-NOP supplementation. Milk FA data from DJ cows were derived from 2 different farms (Table 2). The farm effect showed that milk from farm A had lower concentrations of C18:0, C18:1 *trans*-9, C18:1 *trans*-11, C18:1 *cis*-9, C18:2 n-6 *cis*, C18:3 n-3, and CLA *cis*-9,*trans*-11 than farm B. These changes are possibly due to the different types and proportions of FA in the diet between farms. A treatment × farm effect on the concentrations of C16:0 and C16:1 showed that 3-NOP supplementation increased their concentration in milk from farm A, whereas 3-NOP supplementation decreased them in milk from farm B. The 3-NOP supplementation affected several individual SCFA and MCFA in DJ cows, as the concentrations of C11:0, C13:0, and C15:0 were greater in milk from cows fed 3-NOP diets than control diets. In contrast, the concentrations of C18:0, C18:1 *trans*-11, C18:1 *cis*-9, C18:2 n-6 *cis*, and C18:3 n-3 in milk were lower in cows fed 3-NOP diets compared with control diets. Considering the FA groups, milk from cows fed 3-NOP had greater concentrations of SCFA, and MCFA, and a lower concentration of LCFA than cows fed control diets. The 3-NOP supplementation did not affect the milk FA composition of DR cows.

**Table 1:** Effect of 3-nitrooxypropanol (3-NOP) on fatty acid (FA) composition of bulk milk from Danish Holstein and Danish Red cows in 2 independent studies

Fatty acid^1^ (g/100 g of total FA)	Danish Holstein				Danish Red			
	Control	3-NOP	SEM^2^	*P*-value	Control	3-NOP	SEM^2^	*P*-value
C4:0	3.49	3.73	0.03	<0.001	4.03	4.09	0.04	0.26
C6:0	2.51	2.72	0.02	<0.001	2.82	2.88	0.03	0.15
C8:0	1.48	1.62	0.01	<0.001	1.58	1.63	0.02	0.15
C10:0	3.53	3.91	0.02	<0.001	3.50	3.63	0.06	0.15
C11:0	0.11	0.13	0.00	<0.001	0.10	0.11	0.01	0.16
C12:0	4.01	4.42	0.02	<0.001	4.12	4.26	0.07	0.15
C13:0	0.17	0.19	0.00	<0.001	0.15	0.16	0.01	0.17
C14:0	12.2	12.3	0.04	<0.001	11.7	11.8	0.03	0.28
C14:1	1.01	1.01	0.01	0.66	1.08	1.06	0.01	0.06
C15:0	1.43	1.49	0.00	<0.001	1.24	1.28	0.02	0.17
C16:0	28.2	26.7	0.04	<0.001	31.3	30.9	0.17	0.12
C16:1	1.82	1.73	0.00	<0.001	1.52	1.47	0.02	0.11
C17:0	0.57	0.59	0.00	<0.001	0.54	0.54	0.00	0.12
C18:0	10.5	10.9	0.05	<0.001	10.0	10.1	0.04	0.13
C18:1 *trans*-9	0.70	0.75	0.02	0.12	0.51	0.52	0.00	0.16
C18:1 *trans*-11	1.74	1.67	0.02	0.08	1.17	1.17	0.00	0.41
C18.1 *cis*-9	22.2	21.7	0.08	<0.001	21.0	20.8	0.14	0.15
C18:2 n-6 *cis*	1.73	1.85	0.01	<0.001	1.59	1.60	0.01	0.35
C18:3 n-3	0.65	0.68	0.00	<0.001	0.44	0.44	0.00	0.90
CLA *cis*-9,*trans*-11	0.61	0.56	0.00	<0.001	0.42	0.42	0.00	0.24
SCFA	11.1	12.1	0.09	<0.001	12.0	12.3	0.15	0.14
MCFA	49.7	48.8	0.09	<0.001	51.9	51.7	0.08	0.07
LCFA	39.1	39.0	0.15	0.69	36.0	35.9	0.10	0.26
SFA	68.6	69.1	0.10	<0.01	71.3	71.6	0.15	0.17
MUFA	28.0	27.3	0.09	<0.001	25.8	25.5	0.16	0.13
PUFA	3.38	3.49	0.01	<0.001	2.84	2.86	0.02	0.56

1 Total FA is the sum of the following FA: C4, C6, C8, C10, C11, C12, C13, C14, C14:1, C15, C16, C16:1, phytanic acid, C17, C17:1, C18, C18:1 *trans*-9, C18:1 *trans*-11, C18:1 *cis*-9, C18:2 n-6, CLA *cis*-9,*trans*-11, C18:3 n-3, C18:3 n-6, C20, C20:0 n-6, C20:1, C20:4 n-6, C20:5 n-3, C21, C22, C22:1 n-9, C22:2, C23, C24, and C24:1. Short-chain FA (SCFA; sum of FA with chain lengths <12); medium-chain FA (MCFA; sum of FA with chain lengths ≥12 and <18); and long-chain FA (LCFA; sum of FA with chain lengths ≥18).

2 Standard error of estimated marginal mean.

**Table 2:** Effect of 3-nitrooxypropanol (3-NOP) on fatty acid (FA) composition of bulk milk from Danish Jersey cows in 2 commercial farms

Fatty acid^1^ (g/100 g of total FA)	Farm A		Farm B		SEM^2^	*P*-value		
	Control	3-NOP	Control	3-NOP		Treatment	Farm	Treatment × farm
C4:0	5.90	6.35	6.09	6.43	0.28	0.11	0.56	0.80
C6:0	3.05	2.98	3.06	3.12	0.06	0.63	0.07	0.19
C8:0	1.68	1.62	1.71	1.77	0.02	0.51	<0.01	0.06
C10:0	3.61	3.72	3.76	3.92	0.17	0.48	0.17	0.83
C11:0	0.07	0.10	0.08	0.12	0.01	<0.001	0.14	0.97
C12:0	4.01	4.29	3.98	4.16	0.24	0.18	0.60	0.80
C13:0	0.09	0.12	0.09	0.13	0.01	<0.01	0.12	0.57
C14:0	9.93	10.42	9.96	10.04	0.31	0.18	0.31	0.40
C14:1	0.76	0.83	0.73	0.71	0.07	0.41	0.11	0.41
C15:0	0.93	1.16	0.99	1.14	0.04	<0.001	0.88	0.24
C16:0	32.38	33.68	27.01	26.54	0.42	<0.001	<0.001	0.02
C16:1	1.37	1.42	1.18	1.11	0.03	0.02	<0.001	<0.01
C17:0	0.35	0.34	0.38	0.40	0.01	0.62	<0.001	0.13
C18:0	12.65	11.65	14.22	14.20	0.20	0.01	<0.001	0.21
C18:1 *trans*-9	0.38	0.39	0.41	0.49	0.04	0.33	0.02	0.27
C18:1 *trans*-11	1.04	0.98	1.86	1.67	0.20	0.04	<0.001	0.70
C18.1 *cis*-9	19.31	17.69	21.20	21.06	0.63	0.01	<0.001	0.15
C18:2 n-6 *cis*	1.61	1.46	2.01	1.76	0.08	<0.001	<0.001	0.41
C18:3 n-3	0.25	0.22	0.43	0.41	0.01	<0.001	<0.001	0.67
CLA *cis*-9,*trans*-11	0.29	0.33	0.48	0.45	0.03	0.14	<0.001	0.26
SCFA	14.31	14.76	14.70	15.36	0.28	0.05	0.03	0.65
MCFA	49.99	52.37	44.47	44.37	0.98	<0.01	<0.001	0.12
LCFA	35.71	32.87	40.84	40.28	1.09	<0.01	<0.001	0.20
SFA	74.68	76.42	71.35	71.99	0.61	<0.001	<0.001	0.26
MUFA	23.04	21.40	25.54	25.20	0.61	<0.01	<0.001	0.19
PUFA	2.29	2.18	3.12	2.81	0.11	<0.001	<0.001	0.27

1 Total FA is the sum of the following FA: C4, C6, C8, C10, C11, C12, C13, C14, C14:1, C15, C16, C16:1, phytanic acid, C17, C17:1, C18, C18:1 *trans*-9, C18:1 *trans*-11, C18:1 *cis*-9, C18:2 n-6, CLA *cis*-9,*trans*-11, C18:3 n-3, C18:3 n-6, C20, C20:0 n-6, C20:1, C20:4 n-6, C20:5 n-3, C21, C22, C22:1 n-9, C22:2, C23, C24, and C24:1. Short-chain FA (SCFA; sum of FA with chain lengths <12); medium-chain FA (MCFA; sum of FA with chain lengths ≥12 and <18); and long-chain FA (LCFA; sum of FA with chain lengths ≥18).

2 Standard error of estimated marginal mean.

Although studies exist on determining the effect of 3-NOP on milk FA of Holstein cows (Hristov et al., 2015; Malgar et al., 2020a; Lokuge et al., 2024b), this study is the first to present findings on DJ and DR dairy cows, according to the author's knowledge. The present study showed that 3-NOP supplementation increased the total concentration of SCFA in the milk of both DH and DJ breeds, and similar trends were observed for DR cows without statistical significance. In addition, some MCFA in the milk of DH and DJ cows were increased by 3-NOP. The increased concentration of de novo synthesized FA in milk by 3-NOP supplementation in Holsten dairy cows was in accordance with previous studies (Hristov et al., 2015; Melgar et al., 2021; Lokuge et al., 2024b). It has been suggested that the shift in rumen fermentation toward more butyrate production, and the potential supply of additional energy from lowered CH_4_ emission by 3-NOP may increase de novo synthesis of milk FA, and butyrate is assumed to serve as the major substrate for the de novo synthesis of SCFA under the inhibition of methanogenesis by 3-NOP (Melgar et al., 2020a, 2021). Previous studies including a study conducted under Danish conditions have reported an increased proportion of butyrate in the rumen of Holstein cows due to 3-NOP supplementation (Lopes et al., 2016; Melgar et al., 2020a; Kjeldsen et al., 2024). However, no rumen VFA data were available in our studies to assess the effect of 3-NOP supplementation on rumen fermentation. Nevertheless, 3-NOP significantly reduced CH_4_ emissions from DH, DJ, and DR cows in our studies. The increased concentration of total SFA due to increased production of de novo synthesized SFA in cows fed 3-NOP supplemented diets was consistent with previous studies (Hristov et al., 2015; Melgar et al., 2020b; van Gastelen et al., 2022).

The reduction of the concentration of C16:0 in milk by 3-NOP supplementation in DH cows and in DJ cows from farm B agreed with the studies by van Gastelen et al. (2022) on Dutch Holstein-Friesian cows, and Lokuge et al. (2024b) on DH cows. In the study with DR cows, the concentration of C16:0 was numerically lower due to 3-NOP supplementation, although statistically insignificant. This could be attributed to the lowered de novo synthesis of C16:0 due to a decreased total VFA concentration and a reduced proportion of acetate in the VFA composition when feeding 3-NOP, because previous studies have observed reductions in total VFA and molar proportion of acetate upon 3-NOP supplementation (Haisan et al., 2017; Melgar et al., 2020a; Kjeldsen et al., 2024). However, further studies are required to confirm this hypothesis and to elucidate the underlying biochemical pathways. The interaction effect between farm and treatment on the concentration of C16:0 in milk from DJ cows showed an increase in the concentration of C16:0 by 3-NOP in farm A, in opposite to the decrease in C16:0 observed in farm B. This could be due to differences in the basal diet composition, mainly the type and proportions of FA in the diet. Other farm factors such as health and lactation stage of the cows may also affect the FA composition (Nickerson, 1995). However, we assumed that the difference between farms is mainly driven by differences in diets composition as previous studies reported that the impact of 3-NOP supplementation on milk FA composition depends on the diet composition (van Gastelen et al., 2022; Lokuge et al., 2024b).

In the rumen, ∼70% to 95% of dietary C18:2 n-6 and 85% to 100% of C18:3 n-3 undergo BH, predominantly resulting in the formation of C18:0. During this process, various *trans* FA, such as CLA *cis*-9,*trans*-11 and C18:1 *trans*-11, are produced as intermediate products (Doreau and Ferlay, 1994). The study by Melgar et al. (2020b) suggested that rumen BH may serve as an additional, but minor hydrogen sink under inhibited methanogenesis by 3-NOP. Our study also indicates a potential effect of 3-NOP on BH. However, variability in the observed patterns of change in those FA between DH and DJ cows led to uncertainty, making it challenging to draw a definitive conclusion. The increased concentrations of C18:2 n-6 *cis* and C18:3 n-3 in milk from DH cows fed 3-NOP diets is likely a result of less use of these FA in BH. In contrast, a higher concentration of C18:0 and a lower concentration of CLA *cis*-9,*trans*-11 by 3-NOP diets indicate that the extent of BH may increase by 3-NOP supplementation. The study by Lokuge et al. (2024b) on DH, and the study by van Gastelen et al. (2022) on Dutch Holstein-Friesian cows also suggested that 3-NOP supplementation may affect the degree of rumen BH. In DJ cows, a higher amount of C18:2 n-6 *cis* and C18:3 n-3 may have been directed to the BH process due to lowered concentration of those FA in milk upon 3-NOP supplementation. However, the extent of BH appeared to be reduced in 3-NOP fed cows according to the observed decreases in the concentrations of C18 and C18:1 *trans*-11. The study by van Gastelen et al. (2020) reported a decreasing effect of 3-NOP supplementation on C18:2 n-6 *cis* and C18:3 n-3, but without changes in intermediate or end products of BH in milk. Consistent with previous studies (Hristov et al., 2015; van Gastelen et al., 2022; Lokuge et al., 2024b), 3-NOP reduced MUFA in DH and DJ cows. Contrary to Hristov et al. (2015), Melgar et al. (2021), and van Gastelen et al. (2022), PUFA levels were higher in DH cows on 3-NOP diets. However, 3-NOP decreased the total concentration of PUFA in milk from DJ cows in both farms.

Multivariate data analysis was performed to create a principal component (**PC**) analysis plot (Figure 1). According to the score plot (Figure 1A), there was an obvious breed effect on milk FA composition, whereas 3-NOP supplementation mainly displayed an effect within DH cows and DJ cows in farm A. The first PC, explaining 44% of the variation, separated milk samples from 2 DJ farms from DH and DR milk samples, but with a clear separation also of DH and DR, placing DH samples most to the right and DR close to the center. In contrast, the second PC, which explained 26% of the variation, separated DJ (farm A) and DR from DJ (farm B) and DH. The FA attributed to these differences among the breeds and DJ farms can be identified from the loading plot (Figure 1B). Milk from DJ cows had higher concentrations of saturated SCFA and lower concentrations of UFA than DH cows. Milk from DH cows was characterized by higher concentrations of UFA such as C18:1 *cis*-9; C18:1 *trans*-9; CLA *cis*-9,*trans*-11; and C18:3 n-3. The FA composition of DR cows was intermediate between that of DJ and DR and had higher concentrations of MCFA. These results indicate a clear separation in milk FA composition between breeds, which may reflect underlying genetic differences; however, the potential influence of breed-specific feeding regimens cannot be excluded. In DJ cows, milk FA variation related to the diet composition was displayed. In summary, supplementing 3-NOP in the diet increased de novo synthesized FA in milk from DH, DJ, and DR cows to varying degrees across 3 independent studies. Our results emphasize the importance of further studies to evaluate the interaction effect of 3-NOP with cow breeds on milk FA composition.

**Figure 1:**
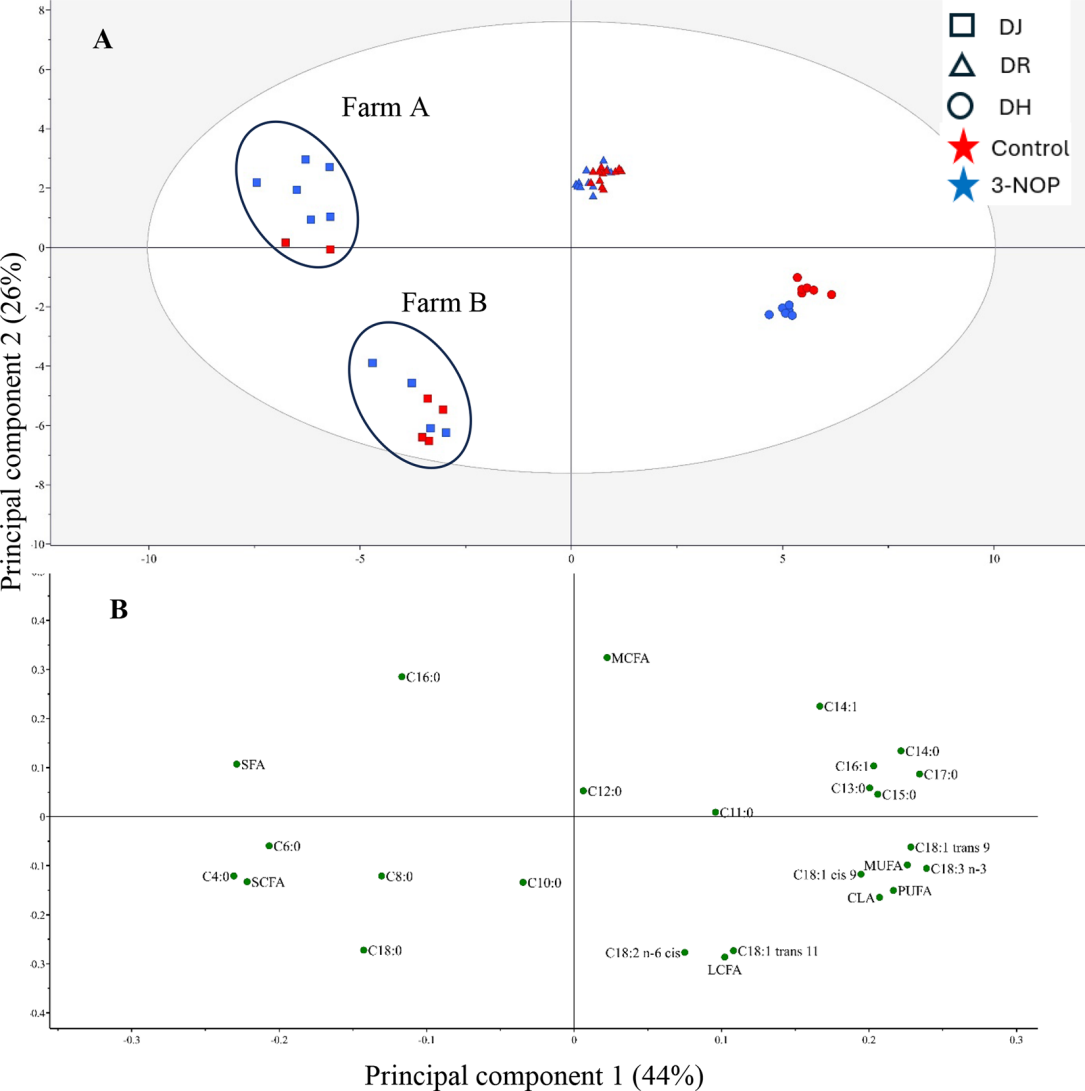
Principal component analysis score plot (A) and loading plot (B) of milk fatty acids of bulk milk as influenced by cow breed (DJ = Danish Jersey; DR = Danish Red; DH = Danish Holstein) and supplementing 3-NOP in the diet at a dose of 60 mg/kg feed DM.
